# Are palpation-guided interventional procedures on the adductor longus muscle safe? A cadaveric and sonographic investigation

**DOI:** 10.1007/s00276-025-03567-2

**Published:** 2025-02-07

**Authors:** Javier Santamaría-Le Pera, Fermín Valera-Garrido, Francisco J. Valderrama-Canales, Francisco Minaya-Muñoz, Pablo Herrero, Diego Lapuente-Hernández

**Affiliations:** 1 Quiron Prevention Health Center, Madrid, 28020 Spain; 2 MVClinic Institute, Madrid, 28600 Spain; 3 CEU San Pablo University, Madrid, 28925 Spain; 4 Invasive Physiotherapy Department, Getafe C.F, Madrid, 28903 Spain; 5 Department of Anatomy & Embryology, Faculty of Medicine, Complutense University of Madrid, Madrid, 28040 Spain; 6 Department of Physiatry and Nursing, Faculty of Health Sciences, University of Zaragoza, Zaragoza, 50009 Spain; 7 iHealthy Research Group, University of Zaragoza/IIS Aragon, Zaragoza, Spain

**Keywords:** Ultrasound, Sonography, Cadaver, Adductor longus muscle, Interventional musculoskeletal procedures

## Abstract

**Purpose:**

The main objective was to study the anatomy of the adductor longus by ultrasound and cadaveric dissection, correlate the findings with the different approaches described, and evaluate the feasibility of defining a “safe window” for interventional musculoskeletal procedures.

**Methods:**

The anatomical study was performed on six cadaveric pieces, while ultrasound evaluations were performed on both lower limbs of 26 subjects (*n* = 52). Ultrasound variables included the number of saphenous veins, the location of the saphenous vein in relation to the proximal myotendinous junction, the number of vessels within or superficial to the adductor longus, and the distance between the dermis and the inferior border of the adductor longus to the anterior branch of the obturator nerve.

**Results:**

Key anatomic risk factors identified in cadavers included the great saphenous vein, the anterior branch of the obturator nerve, and the vascular network traversing the adductor longus. Ultrasound findings revealed that 91.4% of cases had at least one vessel at the proximal myotendinous junction in the cross-sectional area, almost 60% showed two to five vessels within the thickness of the muscle, and the anterior branch of the obturator nerve was located at a mean depth of 3.63–3.93 cm.

**Conclusions:**

It was not possible to define a “safe” approach area without the risk of damaging any neurovascular bundle due to the high anatomical variability both in number and in the route of these along the adductor longus. Therefore, the use of ultrasound to guide any interventional musculoskeletal procedure is highly recommended.

**Supplementary Information:**

The online version contains supplementary material available at 10.1007/s00276-025-03567-2.

## Introduction

The adductor longus muscle is one of the most injured muscles in the hip [11] and therefore is the target of different interventional musculoskeletal procedures, such as dry needling, percutaneous needle electrolysis, percutaneous needle neuromodulation, platelet-rich plasma injection, obturator nerve block and surgery [2, 7, 12, 13, 15], with what this implies in terms of possible compromise of the relevant neurovascular bundles in the area. Before reaching the medial muscular compartment of the thigh, where the adductor longus is located, needles go through the skin, the tela subcutanea -*superficial fascia*-, the fascia lata -the investing fascia of the thigh- and, once into the medial compartment, the adductor longus. On the way to the adductor longus, the needle may reach the great saphenous vein, its tributaries, the external pudendal arteries, the obturator nerve, and even more importantly, all their likely anatomic variants [3].

In the case of the great saphenous vein, three different anatomical variations were described by Dwight et al. in 1907 [8]. One of these is that the great saphenous vein may perforate the fascia lata some distance below the saphenous opening, the second is that it can be occasionally double, and the third is that it can be replaced by a network of veins in which a main trunk is not identified. However, when this region was described with duplex ultrasound by Cavezzi et al. [6], five different variations were described. In this case, the first was that the great saphenous vein ascends in the saphenous compartment (splitting of the fascia lata in which the vein is housed) without large parallel tributaries; the second is that the great saphenous vein comprises two parallel veins in the saphenous compartment; the third is that a single great saphenous vein is located in the saphenous compartment with a large subcutaneous tributary that attaches to it at a variable level in the thigh; the forth is that the great saphenous vein and an anterior accessory saphenous vein join just before the femoral saphenous junction; and the fifth one is that there is a single great saphenous vein in the proximal thigh, in the saphenous compartment, and a large subcutaneous tributary joins it at a variable point along the thigh.

Bergman et al. [3] identified the variants in the tributaries of the great saphenous vein in 1984 describing that in 37% of the cases, the superficial iliac circumflex and the superficial epigastric veins form a common trunk to drain into the great saphenous vein, while the superficial external pudendal vein drains independently before the great saphenous vein enters the saphenous hiatus. In 9% of cases, the anterior accessory saphenous, the superficial iliac circumflex, and the superficial epigastric veins form a common trunk that drains into the saphenous, while also in 9% of the cases, the anterior accessory saphenous and the superficial circumflex iliac veins form a common trunk draining into the saphenous hiatus. Regarding the external pudendal arteries, these can be present in numbers between one to three, the majority being in the presence of two [5, 17].

Another important structure that is relevant when performing interventional procedures is the obturator nerve, which is located deeper into the aforementioned blood vessels. In a recent anatomical study, it was observed that in all the dissected cadavers the anterior branch of the obturator nerve ran anteriorly to the adductor brevis muscle and the posterior branch ran posteriorly to it. On the one hand, the anterior branch runs between the adductor brevis muscle on its ventral side and the pectineus and adductor longus muscles on its dorsal sides, covered throughout its entire length by the fascia of the adductor brevis, and providing numerous muscular branches for the pectineus muscle, the adductor longus, and the adductor brevis. On the other hand, the posterior branch, after emerging from the obturator canal, goes rapidly under the fascia of the adductor magnus muscle and travels in contact with this muscle, giving branches for the adductor brevis and adductor magnus muscles. However, there is a high anatomical variability in the divisions and subdivisions of the obturator nerve, which explains the difficulty frequently found in the application of different techniques, such as regional anesthetics [1].

However, to date, no studies have compared the cadaveric and ultrasound features of the adductor longus and its relationships with neurovascular bundles in the areas most frequently treated with interventional musculoskeletal procedures. The study aimed to investigate if it is possible to define a “safe” entry route (approach window) that avoids needling the superficial vessels as well as determining the average distance between the dermis and the relevant structures in the territory of the adductor longus, describing the anatomy of the adductor longus through cadaveric and ultrasound study and its relationships with the areas most frequently treated within interventional musculoskeletal procedures.

## Materials and methods

### Study design

An observational study was carried out in the period between March and May 2023. In the first stage, a cadaver study was carried out for the recognition of the relevant anatomical structures in the territory of the adductor longus muscle. Subsequently, in a sample of healthy subjects, an ultrasound evaluation was carried out to identify the anatomical structures to study their features and establish possible correlations.

### Study on cadaver

The anatomic study was carried out on cadavers belonging to the Body Donation Center and Dissecting Rooms of the Complutense University of Madrid. The anterior and medial compartments of the thigh from six embalmed lower limbs (4 females and 2 males -ages ranging from 70 to 83-, 3 right and 3 left) were dissected to identify the anatomical structures of interest, to analyze the risks in their approach, and to define the anatomical landmarks that will facilitate its exploration by ultrasound. Corpses were embalmed with Cambridge mixture following the protocol described in Valderrama et al. [18].

### Ultrasound study

26 subjects (*n* = 52; right and left side) without previous pathology, 12 women and 14 men, aged 33.6 ± 10.4 years, participated voluntarily in the present study, after signing an informed consent. A Logiq E R8 ultrasound machine with a 12L-RS linear probe was used, operating at a frequency of 10 MHz. A standardized protocol was defined in the collection of ultrasound images. A total of 52 limbs were evaluated, in a supine position on a table with the backrest positioned at about 45 degrees and with the hips in a comfortable position with slight abduction and external rotation. This position is similar to the FABER maneuver but with less external rotation to increase the tolerance of the participants [14]. The ultrasound probe was placed in a transversal section to the adductor longus muscle to obtain a good visualization of the proximal myotendinous junction at a variable distance between 8 and 12 cm from the insertion of the adductor longus into the body of the pubic ramus. Additionally, the potential impact of probe pressure on the assessment of vascular structures was carefully considered. All examinations were performed with minimal contact, avoiding any exerted pressure beyond what was necessary to maintain proper imaging. The experienced examiner standardized and consistently followed this approach to ensure reliable and accurate evaluations of vascular structures.

**Fig. 1:**
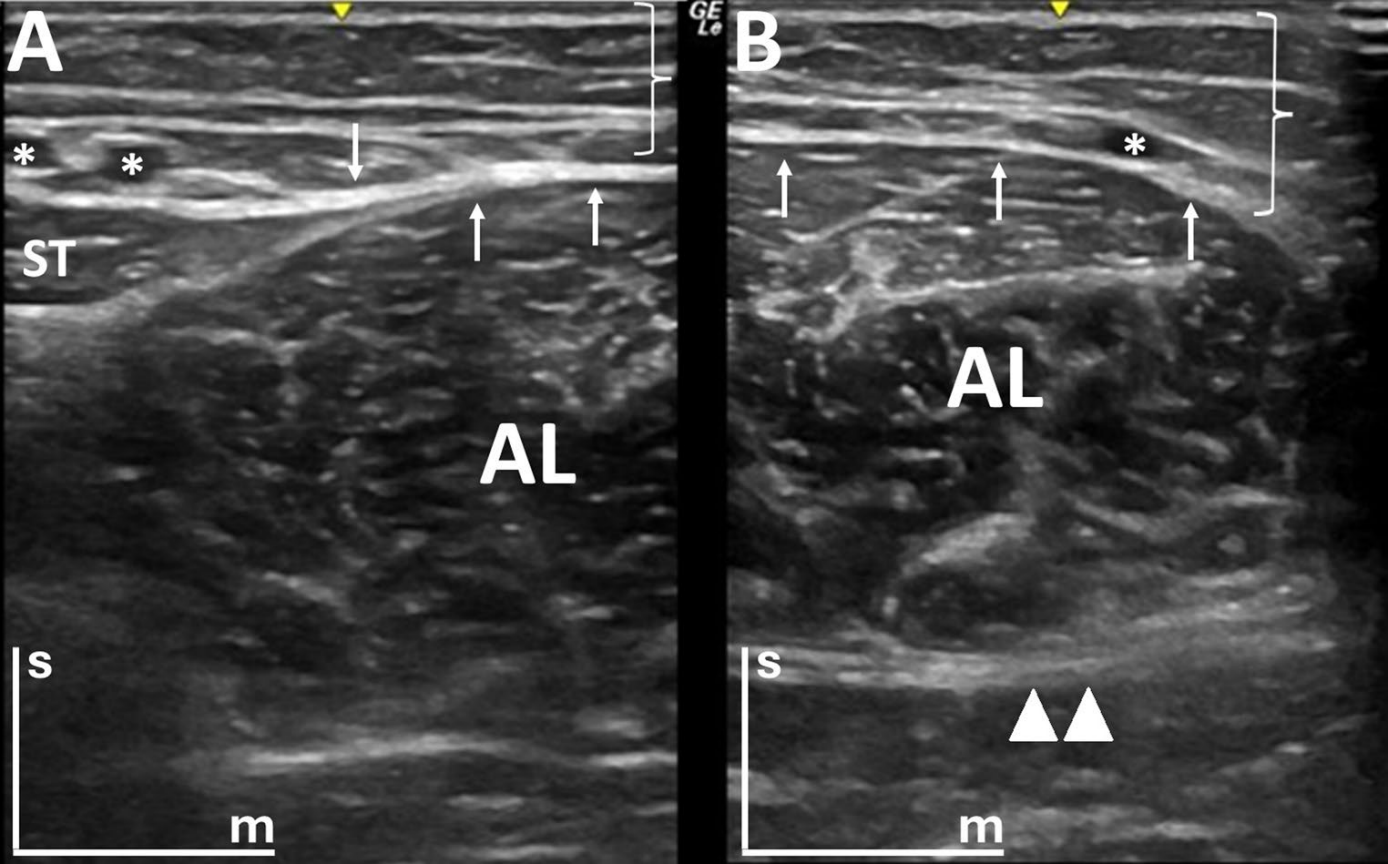
Ultrasound images of the proximal thigh in the transversal section (B-mode). The skin positioned at the top of each image is indicated by the bars (“s” for superficial and “m” for medial). White arrows highlight the fascia lata, and the investing fascia of the thigh. Brackets denote the tela subcutanea (superficial fascia). **A**) This image represents the lateral cut of the adductor longus, where two veins from the great saphenous system are visible (marked with white asterisks); and **B**) This image represents the medial cut of the adductor longus, where only one vein is shown (marked with white asterisks), in addition to the anterior branch of the obturator nerve (represented with a double triangle, ▲▲). AL: adductor longus muscle; ST: sartorius muscle

**Fig. 2:**
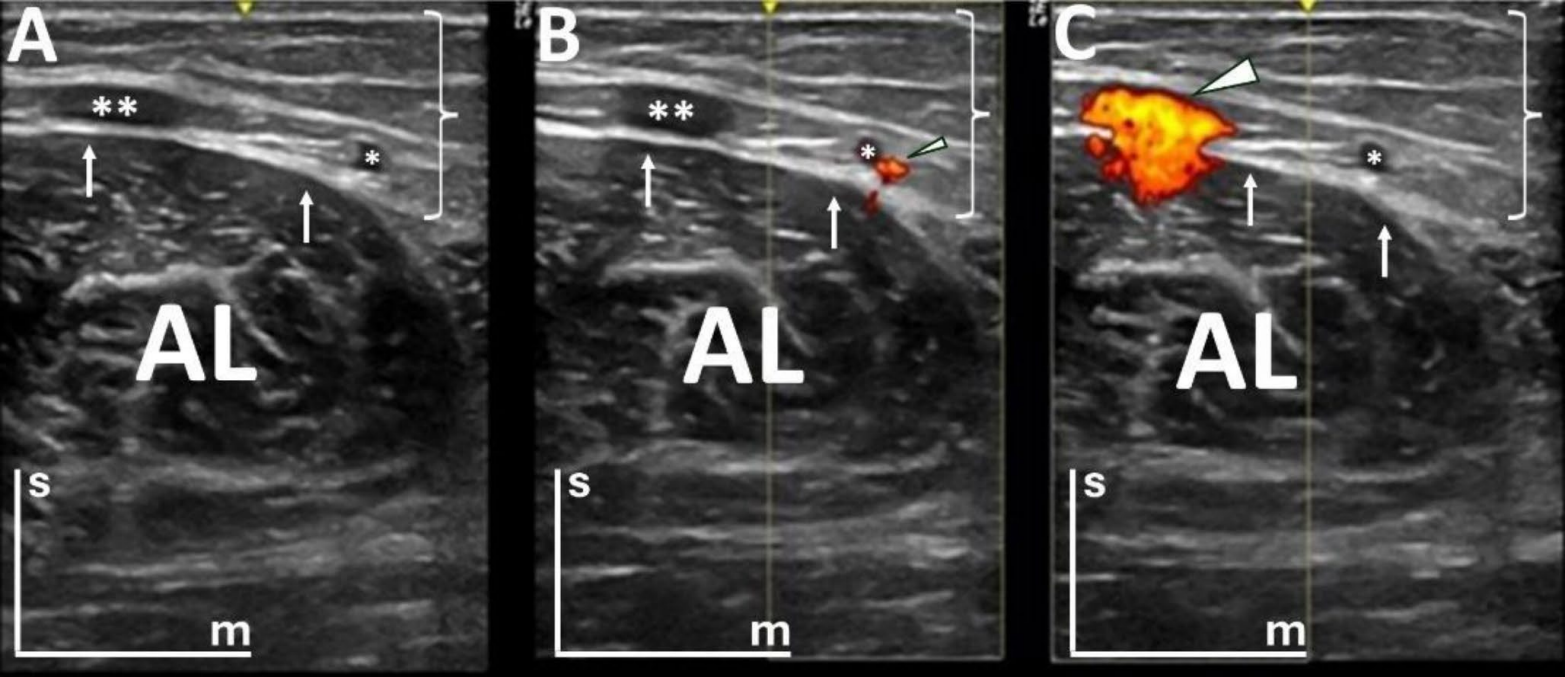
Ultrasound images of the proximal thigh in the transversal section (B-mode and Power Doppler Imaging (PDI) mode). Three images, belonging to the same lower limb, are presented, with the skin positioned at the top of each image, as indicated by the bars (“s” for superficial and “m” for medial). In images B and C, PDI mode is activated, displaying fluid within vascular channels with the Doppler signal in color. White arrows highlight the fascia lata, and the investing fascia of the thigh. Brackets denote the tela subcutanea (superficial fascia). **A**) The great saphenous vein is marked by two asterisks, and a communicating vein is labeled with a single asterisk; **B**) PDI mode focuses on the communicating vein, with blood flow representing the Doppler signal in color (small arrowhead); and **C**) PDI mode highlights the great saphenous vein (large arrowhead). AL: adductor longus muscle

The study parameters were standardized in B-mode and Power Doppler Imaging (PDI) mode. Specifically, the PDI mode evaluation was performed with a frequency of 6.3 MHz, gain set at 20, and pulse repetition frequency (PRF) of 0.8 kHz. In B-mode, the adductor longus muscle of each lower limb was divided into two sections, with one being the image of the lateral cut (Fig. 1A) and the other the image of the medial section for B-mode (Fig. 1B), thus obtaining two cuts in B-mode of each adductor longus. This circumstance was motivated since it was not possible to visualize in a single window the amplitude of the adductor longus muscle. In PDI mode (Fig. 2), for the visualization of blood vessels in each of the sections, the size of the Doppler box was adapted to the middle of the ultrasound screen, with the upper and lower limits allowing the maximum of the screen and the width of the box delimited by the central notch of the ultrasound screen that marks the center of the image. In this way, two images have been taken in PDI mode in each of the cuts in B-mode, always placing the Doppler box in the same sequence. To determine the optimal Doppler box size, preliminary tests were conducted comparing the sensitivity for detecting vascular structures with the dimensions used in the study and a 50% reduction of the box size. No differences were observed between these configurations. Therefore, the chosen box size was standardized to ensure consistency across subjects and avoid potential measurement variations.

To name each image the following cipher has been used: right or left adductor longus, cut “1” or “2” where “1” is lateral and “2” medial, subject number and Doppler with the same sequence in the placement of the box, to the left of the screen first corresponding to lateral of the image in the lower right member and to the right of the screen then corresponding to medial in this case, while in the lower left limb, the Doppler box on the left would correspond to medial and on the right to lateral of the member. Therefore, there were obtained six images of each long adductor, two in B-mode and four in PDI mode.

Based on the anatomical study of cadavers and the bibliographic references, the following outcomes were defined for the ultrasound study:

- Number of saphenous vein/s;
- Location of the saphenous vein/s concerning the proximal myotendinous junction: medial, anterior, anterolateral, or possible combinations (the way the images were divided is explained in Supplementary Information);
- Number of vessels in the thickness of the muscle or superficial to it taking the same references with the proximal myotendinous junction (medial, anterior, and anterolateral);
- Distance from the dermis to the lower limit of the adductor longus muscle was measured to establish the boundary with the anterior branch of the obturator nerve, which courses between the adductor longus and brevis muscles. Measurements were taken in the medial, anterior, and anterolateral zones.

### Statistical analysis

Statistical analysis was performed with SPSS version 25. For each of the variables mentioned above, basic descriptive statistics were carried out (frequency, percentage, valid percentage, and cumulative percentage). Concerning the measurements from the surface of the dermis to the depth of the adductor longus muscle, the mean and standard deviation, as well as the minimum and maximum, were calculated.

## Results

**Fig. 3:**
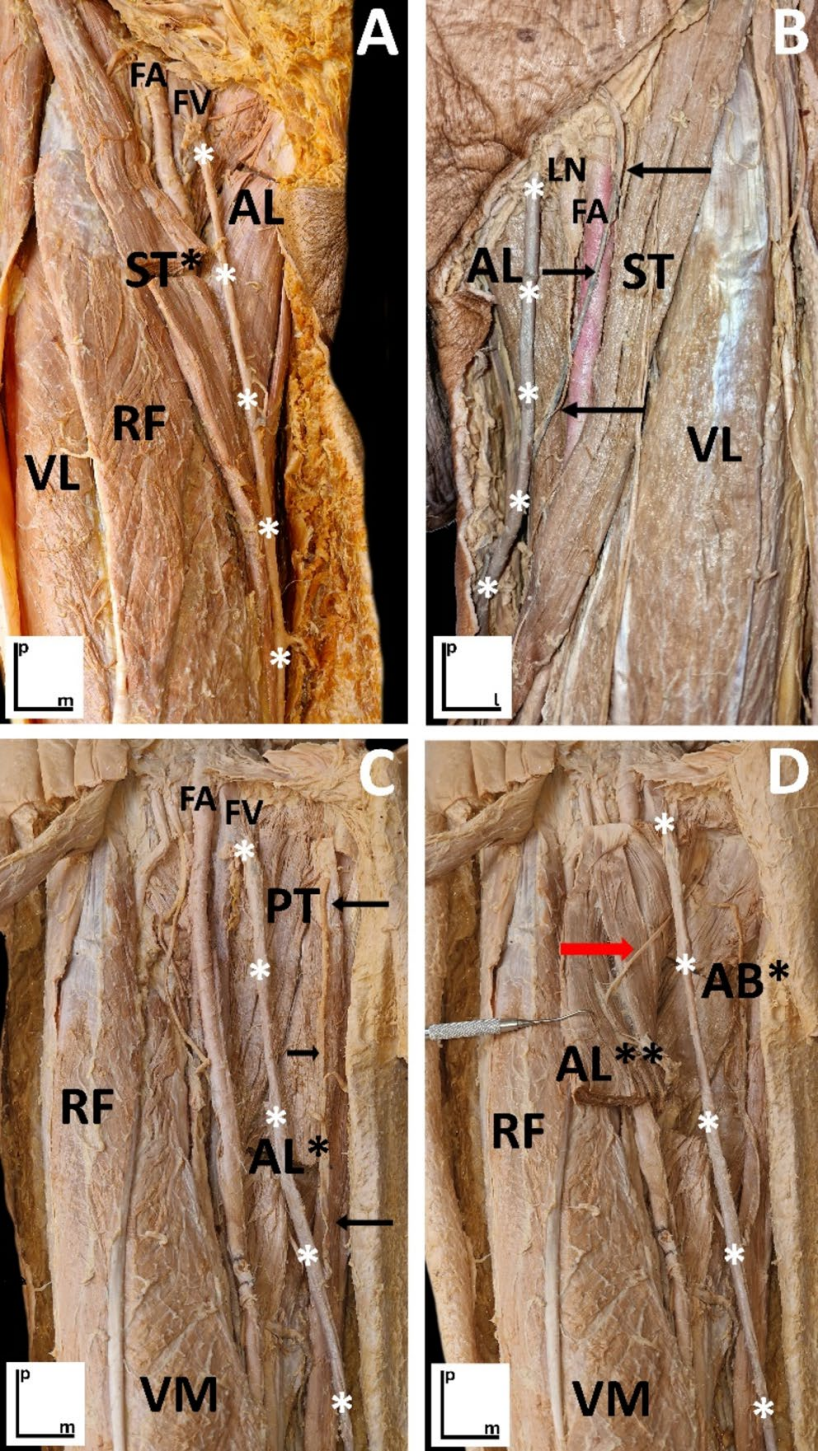
Dissections of the inguinofemoral region, with the fascia lata removed while preserving certain epifascial venous structures. These images highlight the anatomical relationships among the vascular, muscular, and neural structures of the area. Proximal (“p”), medial (“m”), and lateral (“l”) orientations are indicated by the bars. **A**) Right thigh: The great saphenous vein (marked by white asterisks) has been dissected and preserved from the epifascial plane to illustrate its anatomical context within the region. Muscular structures include the sartorius muscle (*cut; ST) adductor longus muscle (AL), vastus lateralis muscle (VL), and rectus femoris muscle (RF). Vascular elements include the femoral artery (FA) and femoral vein (FV); **B**) Left thigh: The great saphenous vein (white asterisks) receives a lateral tributary (black arrows). Key structures shown are the adductor longus muscle (AL), sartorius muscle (ST), vastus lateralis muscle (VL), deep inguinal lymphatic nodule (LN), and femoral artery (FA); **C**) Left thigh: The great saphenous vein (white asterisks) receives a medial tributary (black arrows). The image highlights the pectineus muscle (PT), rectus muscle (RF), and adductor longus muscle (* cut; AL), along with the femoral artery (FA) and femoral vein (FV); and **D**) Left thigh: After cutting and retracting the adductor longus muscle (AL, **) laterally, the anterior branch of the obturator nerve (red arrow) is revealed on its deep surface. Additional structures include the adductor brevis muscle (*cut; AB), rectus femoris muscle (RF), vastus medialis muscle (VM), and the great saphenous vein (white asterisks)

From the anatomical sample, in the subcutaneous layer, the most conspicuous structure was the great saphenous vein that rests on the adductor longus muscle (Fig. 3A and C). At the deep level, the proximal myotendinous junction of the muscle and the anterior branch of the obturator nerve that runs in its deepest superficial plane to the adductor brevis muscle were identified, as well as a network of vessels between the dermis and the thickness of the adductor longus muscle (Fig. 3D).

The main potential risks identified on cadavers with interventional procedures at this level were to damage the great saphenous vein, the anterior branch of the obturator nerve, and the vascular network that crosses the adductor longus muscle (Fig. 3A and D). It should be noted that it was not possible to define any anatomical window that guarantees the palpation-guided approach without risk.

The ultrasound study included 52 thighs (26 right and 26 left), from 26 healthy subjects aged 33.6 ± 10.4 years (12 women). In the total of 52 cases studied, 75% had one saphenous vein, 17.3% had two and in 7.7% the saphenous vein was not visualized at the height of the muscle. In 20.8% the location of the saphenous vein was medial to the proximal myotendinous junction of the adductor longus, in 43.8% it was anterior, and in 16.7% it was anterolateral. In the cases in which two saphenous veins were seen, there were different locations for the proximal myotendinous junction (medial plus anterior, medial plus anterolateral, or anterior plus anterolateral) (Table 1).

**Table 1:** Location of the saphenous vein with respect to the proximal myotendinous junction

Saphenous veins location	Frequency	Percentage	Valid percentage	Cumulative percentage
Medial	10	19.2	20.8	20.8
Anterior	21	40.4	43.8	64.6
Anterolateral	8	15.4	16.7	81.3
Medial + anterior	4	7.7	8.3	89.6
Medial + anterolateral	1	1.9	2.1	91.7
Anterior + anterolateral	4	7.7	8.3	100.0
Lost (not located)	4	7.7	-	-

In addition to the saphenous vein, different vessels have also been counted. Between the dermis and the deepest limit of the muscle, two anterolateral vessels were found in 5.8% of cases, one anterolateral vessel in 42.3% of cases, and no anterolateral vessel in 51.9% of cases. In the case of the anterior vessels, there was a great variety, with three vessels (1.9%), two vessels (7.7%), one vessel (61.5%) and no vessel (28.8%). Finally, in the case of medial vessels, there were two (3.8%), one (44.2%), and none (51.9%). Regarding the total number of vessels in their anterolateral, anterior, and medial locations, it should be noted that 91.4% had at least one vessel and that 59.6% had between two and five vessels in the region (Table 2).

**Table 2:** Total number of vessels (medial, anterior, and anterolateral zones)

Total numbers of vessels	Frequency	Percentage	Valid percentage	Cumulative percentage
0	5	9.6	9.6	9.6
1	16	30.8	30.8	40.4
2	18	34.6	34.6	75.0
3	7	13.5	13.5	88.5
4	5	9.6	9.6	98.1
5	1	1.9	1.9	100.0

Regarding the distance between the surface of the dermis to the depth of the adductor longus muscle in its anterolateral, anterior, and medial zone with the anterior branch of the obturator nerve, the mean distance ranged from 3.63 to 3.93 cm (Table 3).

**Table 3:** Average distance in cm from the surface of the dermis to the lower limit of the adductor longus muscle, measured to define the distance to the anterior branch of the obturator nerve

Zones	Minimum	Maximum	Mean	Standard deviation
Medial	2.28	4.98	3.63	0.69
Anterior	2.65	4.83	3.84	0.61
Anterolateral	2.70	4.93	3.93	0.57

## Discussion

The cadaveric and ultrasound evaluation of the different neurovascular bundles around the area of the adductor longus muscle shows that 91.4% of the sample (47 cases) presented at least a vessel in the transversal section at the height of the proximal myotendinous junction, where acute and chronic muscle injuries are frequent and therefore a vessel can be potentially reached with when an interventional procedure is performed at this level. The variability observed in the number and distribution of blood vessels in the area of the adductor longus (almost 60% had between two and five vessels in the thickness of the adductor longus) prevents the establishment of a “safe” window for the palpation-guided approach with anatomical references. In this sense, it is essential for safety and to avoid adverse effects such as hematoma, that interventional musculoskeletal procedures are performed ultrasound-guided.

Regarding the obturator nerve, it is important to note that at a mean depth of 3.63–3.93 cm., just before the thickness of the adductor longus, the anterior branch of this nerve is located, and it could also be damaged when performing interventional musculoskeletal procedures at this level. Although there is no extensive literature about interventional musculoskeletal procedures, it is frequent that the obturator nerve is treated with an injection of local anesthetics and/or percutaneous needle neuromodulation, which are recommended to be performed ultrasound-guided to locate the nerve precisely and to reduce the rates of vascular puncture [1, 9]. This nerve has been the subject of several cadaveric studies because of its varied anatomic locations and the presence of accessory branches [20]. The first study in which ultrasound visualization of this structure was carried out to improve regional block in clinical anesthesia was that of Soong et al. in 2007 [16]. In this study, the mean distance from the ultrasound transducer to the pubic tubercle when visualizing the anterior and posterior obturator nerve branches in parallel was 2.1 ± 1.2 cm laterally and 2.1 ± 2.0 cm distally. The common obturator nerve and the posterior division were found deeper (25.9 ± 7.6 and 29.3 ± 7.9 mm, respectively) than the anterior division (15.5 ± 3.9 mm).

There is now growing interest in the analysis of adverse effects found after interventional musculoskeletal procedures. The study of Brady et al. [4] about deep dry needling reports a rate of 19.8% of adverse effects. The common ones described were hematoma (7.55%), bleeding (4.65%), pain during treatment (3.01%), and post-needling pain (2.19%). The high variability of the vascular distribution around the adductor longus muscle is a potential factor of adverse effects if the approach is not ultrasound-guided. Although nerves do not present as much anatomical variability as arteries and veins, it is possible to damage a nerve during an interventional musculoskeletal procedure [10, 19].

One of the study’s strengths is its clinical orientation, as the territory of the proximal myotendinous junction of the adductor longus muscle was selected since, according to the authors ´ clinical experience, it is the most prevalent injured area. We have not identified any studies that have described such a circumstance. However, the study also presents some limitations that must be considered when interpreting the findings. First, the ultrasound section performed exclusively at the proximal myotendinous junction makes it impossible to describe the tributary veins and the external pudendal artery defined in the scientific literature [3,5,6,17]. To overcome this limitation, a study including a sweep from proximal to distal should be performed to fully describe the region. Furthermore, the dissection images do not aim to establish a direct correlation with the findings from cadaver ultrasound scans, as this would require a larger anatomical sample or an expanded study design that integrates additional dissection. Additionally, the use of large Doppler boxes may have underestimated the number of vessels and their precise location. While this approach ensures the capture of all potential vessels along the needle trajectory for clinical safety during interventional musculoskeletal procedures, adapting the Doppler boxes more specifically to the structures of interest could enhance anatomical precision. Lastly, another limitation was that the fatty volume of the area analyzed hindered the visualization of the neurovascular bundles. Therefore, recommendations for future studies include the definition of different subgroups based on body mass index or body fat percentage to overcome this limitation and using different cuts along the muscle to see the different routes of the vessels, as well as taking advantage of the ultrasound to perform a sweep that allows to see where the vessels run throughout their journey, thus allowing to describe approach windows at the level of the proximal and distal myotendinous junction, as well as in the muscular belly.

## Conclusions

There was a high variability in the number and location of vascular bundles (superficial and deep) in the territory of the adductor longus, which impedes defining a “safe” approach window for palpation-guided interventional musculoskeletal procedures only with anatomical references. In terms of nerve structures, there does not seem to be as much variability and the risk appears after exceeding a depth of more than four centimeters. Therefore, the ultrasound-guided approach would be the only safe way to perform interventional techniques at this level.

## Electronic supplementary material

Below is the link to the electronic supplementary material.

Supplementary Material 1

## Data Availability

No datasets were generated or analysed during the current study.

## Declarations

### Informed content

All subjects who participated in the study signed an informed consent form.
