# Supplementary material for: Are palpation-guided interventional procedures on the adductor longus muscle safe? A cadaveric and sonographic investigation
Source: Surg Radiol Anat. 2025 Feb 7;47(1):74. doi: 10.1007/s00276-025-03567-2 (PMC11805763; doi:10.1007/s00276-025-03567-2)

**Are palpation-guided interventional procedures on the adductor longus muscle safe? A cadaveric and sonographic investigation**

**Journal: Surgical and Radiologic Anatomy**

**Javier Santamaría^a^, Fermín Valera-Garrido^b,c,d^, Francisco J Valderrama-Canales^e^, Francisco Minaya-Muñoz^b,c^, Pablo Herrero^f,g^, Diego Lapuente-Hernández^f,g^**

**^a^** Quiron Prevention Health Center, 28020 Madrid, Spain

**^b^** MVClinic Institute, 28600 Madrid, Spain

**^c^** CEU San Pablo University, 28925 Madrid, Spain

**^d^** Invasive Physiotherapy Department, Getafe C.F., 28903 Madrid, Spain

**^e^** Department of Anatomy & Embryology. Faculty of Medicine. Complutense University of Madrid, 28040 Madrid, Spain; [fvalderr@ucm.es](mailto:fvalderr@ucm.es) (0000-0002-4288-2940)

**^f^** Department of Physiatry and Nursing, Faculty of Health Sciences, University of Zaragoza, 50009 Zaragoza, Spain; [pherrero@unizar.es](mailto:pherrero@unizar.es) (0000-0002-9201-0120); [d.lapuente@unizar.es](mailto:d.lapuente@unizar.es) (0000-0002-6506-6081)

**^g^** iHealthy Research Group, University of Zaragoza/IIS Aragon, Zaragoza, Spain**.**

*** Correspondence to:** Department of Physiatry and Nursing, Faculty of Health Sciences, University of Zaragoza, 50009 Zaragoza, Spain; iHealthy Research Group, University of Zaragoza/IIS Aragon, Zaragoza, Spain; [pherrero@unizar.es](mailto:pherrero@unizar.es)

**SUPPLEMENTARY INFORMATION**

Anterolateral, anterior and medial zone in relation to the proximal myotendinous junction of adductor longus

- Right adductor longus


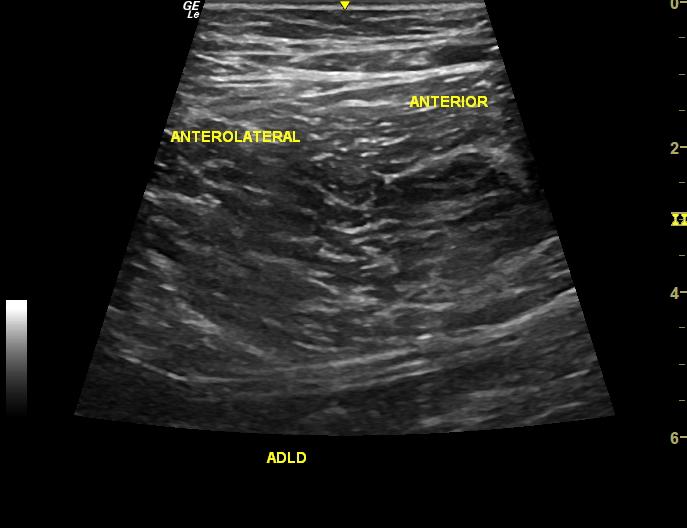

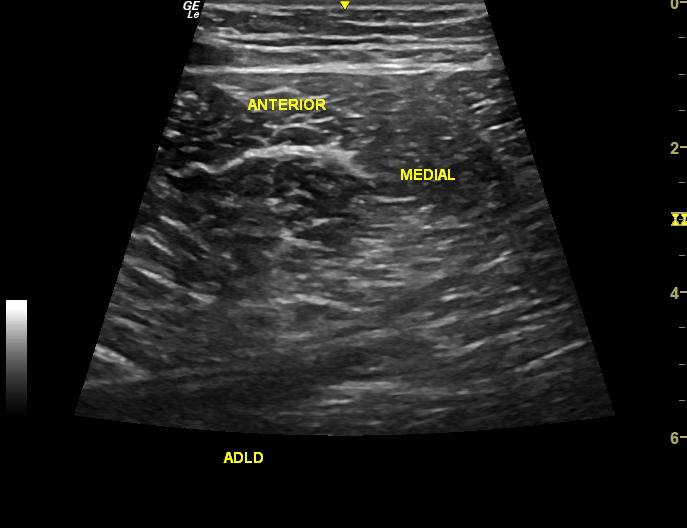


- Left adductor longus


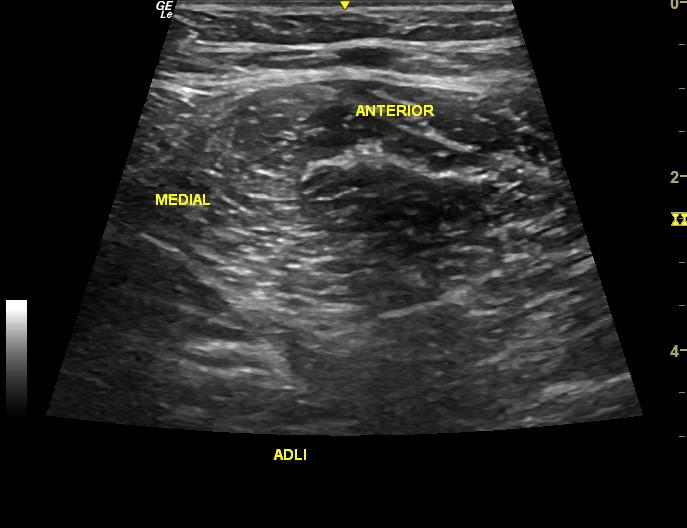

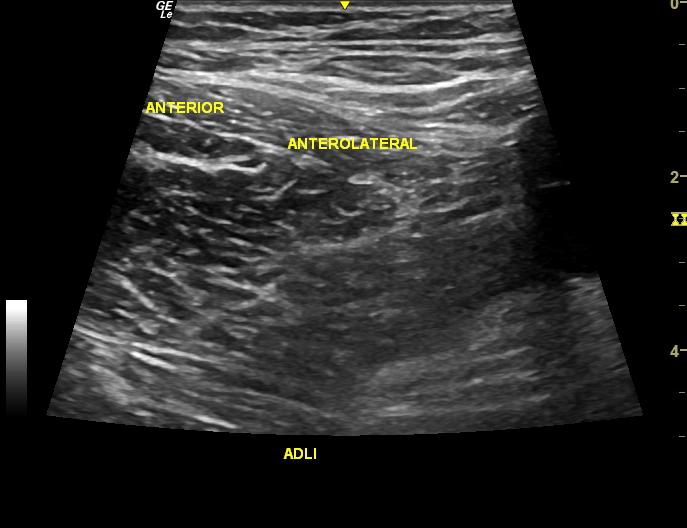

Supplement: Supplementary file 1 — Supplementary Material 1 [file 276_2025_3567_MOESM1_ESM.docx]
